# Vitiligo associated with ribociclib therapy: a rare case report

**DOI:** 10.3389/fonc.2025.1669407

**Published:** 2025-12-08

**Authors:** Zirui Wang, Yuanrui Bai, Rihan Wu, Yihui Liu, Chunhui Dong, Ling Chen

**Affiliations:** 1 Department of Oncology, The First Affiliated Hospital of Xi’an Jiaotong University, Xi’an, China; 2 Health Science Center, Xi’an Jiaotong University, Xi’an, China; 3 Radiotherapy Oncology, People’s Hospital of Ningxia Hui Autonomous Region, Yinchuan, China; 4 Cardiovascular Hospital, Ninth Hospital of Xi’an, Xi’an, China

**Keywords:** ribociclib, vitiligo, CDK4/6 inhibitor, breast cancer, immune-related adverse event

## Abstract

**Background:**

Ribociclib, a cyclin-dependent kinase 4/6 (CDK4/6) inhibitor, occupies a pivotal role in the clinical management of metastatic breast cancer, with its hematologic and gastrointestinal toxicities well-established. However, cutaneous adverse reactions induced by this agent—among which vitiligo is particularly prominent—are extremely rare. Herein, we report a case of vitiligo-like lesions induced by ribociclib, aiming to elucidate this underrecognized drug-adverse reaction association and conduct an in-depth analysis of its potential immune-mediated mechanism.

**Case summary:**

A 59-year-old woman with ER/PR (90% strong+), HER2-negative metastatic breast cancer developed pruritic hypopigmented patches on her forearms 16 months after initiating ribociclib/letrozole. Dermoscopy revealed complete pigment loss, peripheral hyperpigmentation, and telangiectasia. Wood’s lamp examination demonstrated bright blue-white fluorescence, confirming non-segmental vitiligo. No personal/family history of autoimmunity was noted. Topical 0.1% mometasone cream and 0.1% tacrolimus ointment were initiated, with stabilization of existing lesions and no new depigmentation at 2-month follow-up. Ribociclib was maintained due to ongoing tumor control.

**Conclusions:**

HR-positive metastatic breast cancer patients using ribociclib may develop vitiligo, with individual differences (e.g., over one-year incubation as in this case). Combined with prior reports, it suggests a link between ribociclib and vitiligo. Further clinical observations and studies are needed to confirm causality, and pre-use risk explanations to patients are advisable.

## 1 Introduction

Hormone receptor-positive/human epidermal growth factor receptor 2-negative (HR+/HER2−) is the most common molecular subtype of breast cancer (1). In this subtype, dysfunction of cyclin-dependent kinases 4/6 (CDK4/6)—key proteins regulating the transition of the cell cycle from G1 to S phase—is widespread and can drive tumor proliferation. Dysregulation of CDK4/6 has been observed in most hormone receptor-positive (HR+) breast cancers (2). CDK4/6 inhibitors are a new generation of therapeutic compounds capable of binding to DNA and proteins and exhibiting anticancer activity. Their core mechanism of action involves blocking the mitotic cycle by inhibiting CDK4/6. Studies have further confirmed that CDK4/6 selective inhibitors and estrogen receptor (ER) antagonists exhibit significant synergistic effects in inhibiting the proliferation of ER-positive breast cancer cells (3). This mechanistic insight jointly led to the clinical strategy of combining CDK4/6 inhibitors with estrogen antagonists, which has yielded positive clinical treatment outcomes (4).

As a selective CDK4/6 inhibitor, ribociclib delays cancer progression by inhibiting CDK4/6. This drug is a pyrimidine derivative, appearing as a white, odorless, crystalline lipophilic powder. Research using voltammetry, spectrophotometry, and molecular docking techniques has confirmed that its molecule interacts with DNA through minor groove binding (5). Based on its mechanism of action, ribociclib holds an important position in the treatment of HR-positive breast cancer and is now widely used as first-line therapy for advanced breast cancer and as adjuvant therapy for early breast cancer.

Several key clinical studies have provided solid evidence for its clinical application:

1. Evidence for First-Line Advanced Therapy (MONALEESA-2): As a Phase III trial, it evaluated the efficacy and safety of ribociclib combined with letrozole as a first-line regimen in postmenopausal patients with HR-positive, HER2-negative advanced breast cancer. Analyses showed that the ribociclib plus letrozole group had significantly better progression-free survival than the placebo plus letrozole group (updated median analysis: 25.3 months vs. 16.0 months; hazard ratio for disease progression or death, 0.57, P < 0.001) (6, 7). After a median follow-up of 6.6 years, the combination regimen continued to demonstrate a significant overall survival benefit, with median overall survival of 63.9 months in the ribociclib group versus 51.4 months in the control group (hazard ratio for death, 0.76) (8). This indicates that ribociclib combination therapy significantly reduced the risk of death by 24% and extended median overall survival by over one year.

2. Evidence for Treatment of Aggressive Disease (RIGHT Choice): This Phase II study compared first-line ribociclib combined with endocrine therapy versus combination chemotherapy in premenopausal patients with aggressive HR+/HER2- advanced breast cancer, including those with investigator-assessed visceral crisis. Results showed that the median progression-free survival in the ribociclib combination therapy group was 21.8 months, significantly better than the 12.8 months in the chemotherapy group (hazard ratio, 0.61; P = 0.003). Furthermore, the ribociclib group demonstrated superior overall response rates (66.1% vs. 61.8%) and median time to response (4.9 months vs. 3.2 months), along with lower rates of symptomatic adverse events and fewer treatment discontinuations due to treatment-related adverse events (9).

3. Evidence for Early Adjuvant Therapy (NATALEE): This global, multicenter, randomized controlled Phase III study aimed to evaluate the effect of ribociclib combined with an aromatase inhibitor as adjuvant therapy in patients with high-risk HR+/HER2- early breast cancer. The 4-year outcomes presented at the 2024 ESMO Annual Meeting showed that after a median follow-up of 44.2 months, compared with the non-steroidal aromatase inhibitor (NSAI) alone group, the ribociclib plus NSAI group had a significantly improved invasive disease-free survival rate (4-year rate: 88.5% vs. 83.6%) and reduced the risk of invasive disease recurrence by 28.5%.

Based on this robust clinical evidence, the National Comprehensive Cancer Network Clinical Practice Guidelines have recommended the ribociclib adjuvant treatment regimen as a Category 1 preferred treatment option for patients with high-risk HR+/HER2- early breast cancer, thereby establishing its standard position in the comprehensive management of breast cancer throughout the disease course.

## 2 Case illustration

A 59-year-old female patient underwent radical resection of right breast cancer in a local hospital seventeen years ago, and the relevant indicators were normal in the annual re-examinations after the operation. Forty days ago, in May 2023, she visited the Department of Orthopedics of the First Affiliated Hospital of Xi’an Jiaotong University due to right lower limb pain. The CT image diagnosis on May 31, 2023 showed: 1. Multiple nodules and micronodules in both lungs, which were highly suspected to be metastatic tumors. 2. Bone destruction of the manubrium sterni with slightly low-density soft tissue shadow inside; multiple slightly low-density masses were seen in the sternal running area and on both sides, the larger one was located under the xiphoid process, and bone destruction was found on the right side of the S1-S2 vertebrae, which were considered as metastatic tumors. Magnetic resonance imaging showed multiple ring-enhancing lesions in segment S4 of the liver, suggesting metastasis. She was diagnosed with multiple metastases in the sacrum, sternum and liver, pulmonary metastases to be further examined, and lumbar disc herniation. On June 2, 2023, she received spinal canal decompression. Pathology showed: “A small piece of papillary adenocarcinoma tissue from the sacral vertebrae, and combined with the medical history and immunohistochemical staining results, it suggested breast cancer metastasis, with ER (strongly positive, 90%), PR (strongly positive, 90%), HER2 (2+), and Fish (-).

In July 2023, she started to take oral ribociclib succinate tablets 600 mg once a day and letrozole 2.5 mg a day. After treatment, the tumor markers CEA decreased from 11.7 ng/ml to 6.54 ng/ml, CA125 from 46.2 U/ml to 5.2 U/ml, and CA153 from 182 U/ml to 26.2 U/ml. The xiphoid mass reduced from 36×37×32 mm to 18×16×16 mm, and the maximum diameter of liver lesions decreased from 29 mm to 18 mm.

**Figure 1:**
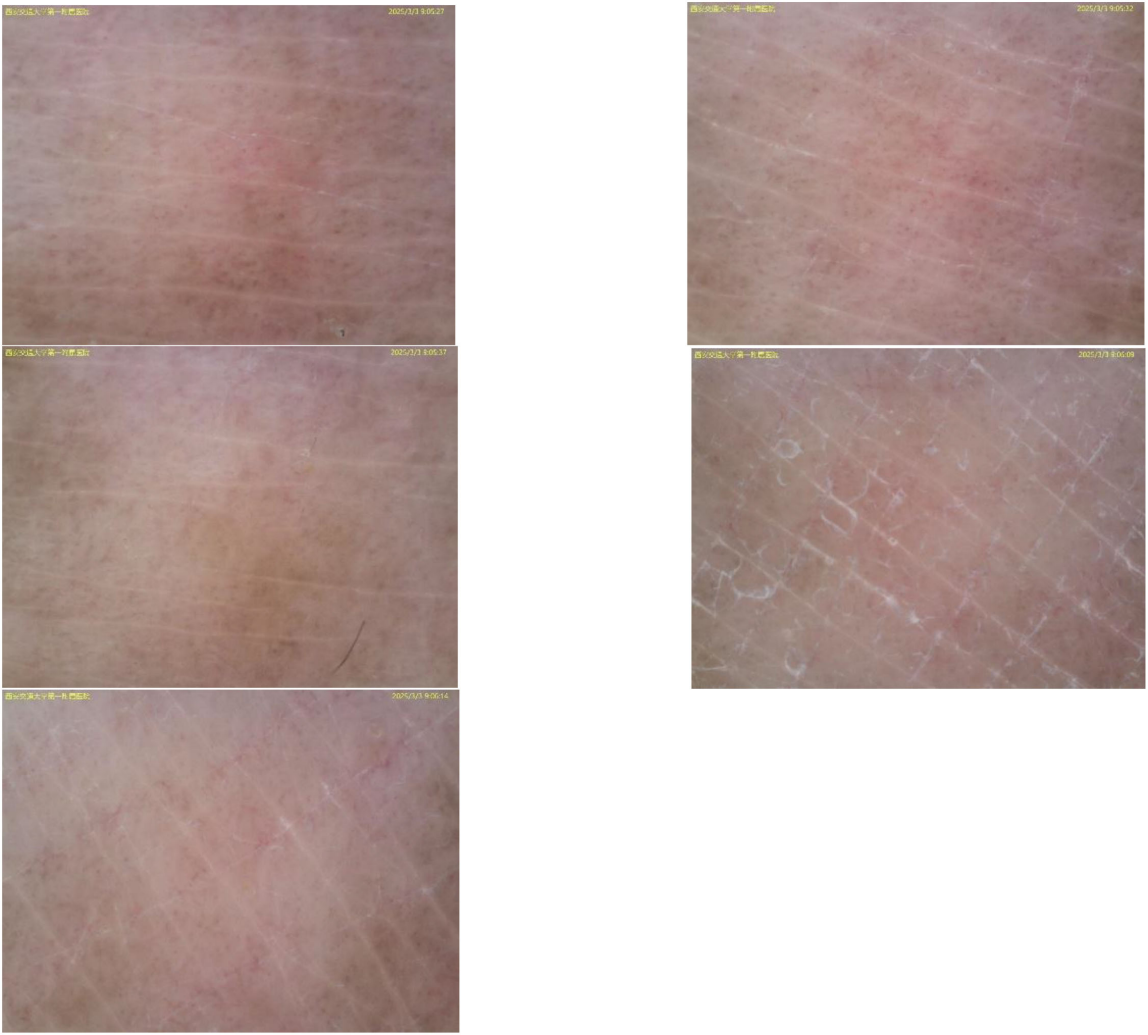
Dermoscopy showed: depigmentation in the lesion area with clear borders, obvious contrast due to pigment deepening around the lesion, and telangiectasia within and around the area, consistent with the typical manifestations of vitiligo.

**Figure 2:**
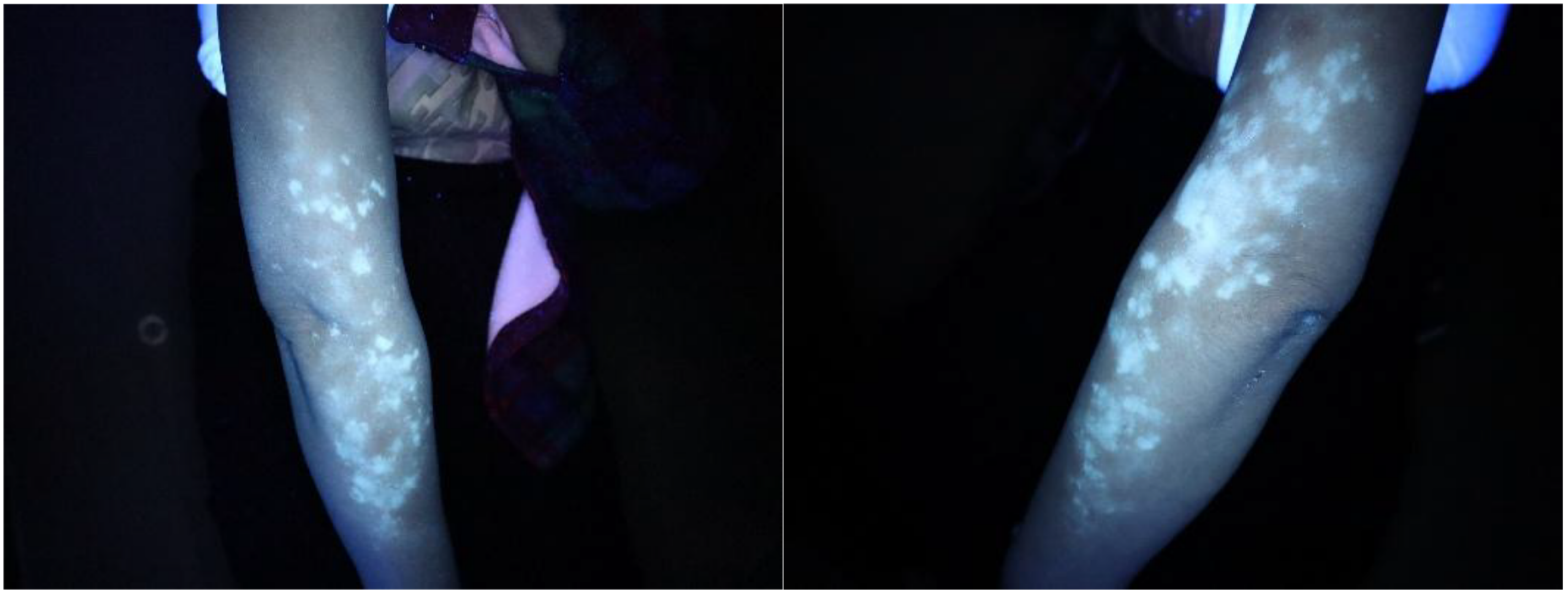
Under Wood’s lamp, the lesion area showed bright blue-white fluorescence, which was in significant contrast with the surrounding normal skin, consistent with the characteristic manifestations of vitiligo.

Sixteen months after the start of treatment, pruritus appeared on the forearm, accompanied by depigmented spots. The patient herself and her family had no previous history of autoimmune diseases. Dermoscopy showed: pigment disappearance in the lesion area, deepened pigment around the lesion, and telangiectasia, suggesting vitiligo (see **Figure 1**). Immunofluorescence and Wood’s lamp examination showed bright blue-white fluorescence(see **Figure 2**). She was treated with 0.1% mometasone cream and 0.1% tacrolimus ointment. The follow-up after 2 months showed stable condition, and no new lesions appeared in other parts.

## 3 Discussion

Three CDK4/6 inhibitors with similar mechanisms of action—palbociclib, ribociclib, and abemaciclib—have received international approval, though their safety profiles exhibit significant differences. As a representative agent of CDK4/6 inhibitors, ribociclib demonstrates common adverse reactions including hepatotoxicity (10), neutropenia, thrombocytopenia, QT interval prolongation, gastrointestinal disturbances such as nausea, diarrhea, and vomiting, along with fatigue (11). Cutaneous adverse events primarily manifest as eczematous dermatitis and maculopapular reactions (12), while vitiligo remains exceptionally rare. It is particularly noteworthy that no vitiligo cases were explicitly reported in the phase III MONALEESA-2, -3, and -7 trials (6–8, 13, 14).

Vitiligo represents an acquired depigmenting disorder affecting cutaneous and mucosal surfaces, potentially involving hair follicles, and demonstrates global epidemiological distribution. Although its precise etiology remains incompletely elucidated, emerging evidence confirms an association between CDK4/6 inhibitor therapy and vitiligo development (15).

In the present case, the patient had no documented history of autoimmune diseases, familial predisposition, or chemical exposure, having received ribociclib solely for breast cancer treatment. Vitiligo was diagnosed via Wood’s lamp examination 16 months post-treatment initiation. Of particular significance, the latency period in this case (16 months) substantially exceeds previously reported intervals (e.g., 8 months (16), 2 months (17)), suggesting that CDK4/6 inhibitors may potentially induce delayed cutaneous toxicity necessitating extended monitoring periods. From an oncological perspective, the patient maintained therapeutic response to ribociclib at vitiligo onset (16 months after treatment initiation) with satisfactory disease control. Current literature reveals heterogeneous outcomes following drug discontinuation, including both disease progression and prolonged stability, though the underlying mechanisms remain undefined. For manageable cutaneous reactions, treatment continuation may be considered, whereas severe cases might warrant temporary suspension of CDK4/6 inhibitors with transition to endocrine monotherapy. In this specific case, although vitiligo manifestations occurred, the lesion areas showed no further expansion without compromising life quality, and the original lesions continued to regress. Following comprehensive risk-benefit discussion, the patient elected to continue the ribociclib-letrozole combination therapy.

The core pathogenesis of vitiligo involves CD8+ T cell-mediated specific immune destruction of melanocytes (18). From a pharmacodynamic perspective, ribociclib-induced vitiligo may occur through multiple pathways: the agent significantly inhibits the immunoregulatory function of regulatory T cells (Treg), leading to loss of normal immune surveillance and subsequent autoreactive CD8+ T cell-mediated targeting of melanocytes; *in vitro* experiments confirm that CDK4/6 inhibitors can directly arrest melanocyte cycle progression and induce programmed cell death (19). Notably, recent investigational evidence indicates that short-term exposure to CDK4/6 small molecule inhibitors can significantly enhance T cell activation through derepression of the transcriptional suppression state of Nuclear Factor of Activated T Cells (NFAT) family proteins, a mechanism that partially explains the *in vivo* antitumor effects of these agents (20). Although CDK4/6 inhibitors demonstrate inhibitory effects on T cell proliferation *in vitro*, they promote effector T cell activation and tumor tissue infiltration within the tumor microenvironment (20). This mechanism shares similarities with phenomena observed in immune checkpoint inhibitor therapy. In immunotherapy for metastatic melanoma, vitiligo emergence is regarded as a characteristic immune-related adverse event, occurring in 15% of patients and showing significant correlation with favorable treatment response (21). Based on this evidence, we hypothesize that while activating antitumor immune responses, CDK4/6 inhibitors may disrupt autoimmune tolerance toward melanocytes, consequently triggering vitiligo development. Furthermore, dermatoscopic observations reveal localized VEGF signaling pathway activation accompanied by capillary dilation phenomena in lesions, suggesting potential involvement of vascular microenvironment alterations in disease pathogenesis. Particularly noteworthy is that compared to other CDK4/6 inhibitors, ribociclib demonstrates superior blood-brain barrier permeability, and this unique pharmacokinetic characteristic might exert more pronounced effects on the cutaneous neuro-immune-melanocyte regulatory network, a hypothesis requiring urgent validation through subsequent investigations.

Our analysis of 12 reported CDK4/6 inhibitor-associated vitiligo cases identifies ribociclib as the predominantly involved agent (83.3%, 10/12), with palbociclib and abemaciclib each accounting for 8.3% (1/12 respectively) (**Table 1**). It must be specifically emphasized that the incidence of cutaneous manifestations varies among different CDK4/6 inhibitors, potentially attributable to differences in their respective mechanisms of action. The median patient age was 71.5 years, with vitiligo onset occurring between 2 to 11 months post-CDK4/6 inhibitor treatment. Following vitiligo presentation, the following management approaches were implemented: 1) Switching to palbociclib after vitiligo development during ribociclib treatment resulted in dermatological symptom recurrence, indicating that alternative CDK4/6 inhibitors cannot alleviate vitiligo-related cutaneous damage ^[21(1),22]^; 2) Six-month ribociclib discontinuation led to disease progression, necessitating treatment reinitiation with subsequent maintained lesion stability ^[21(1)]^; 3) Drug cessation resulted in sustained complete metabolic response ^[21(2)23]^; 4) Dose reduction achieved concurrent stability in both oncological status and vitiligo (17); 5) Continued original treatment for mild lesions showed no disease progression (24, 25); 6) Oral mini-pulse betamethasone (5 mg for two consecutive days weekly), levocetirizine (5 mg nocturnally), and emollient therapy successfully arrested further depigmented lesion progression (16, 26–28).

**Table 1:** Cases of vitiligo in patients after using CDK4/6 inhibitors.

Cdk4/6i	No	Age (y)	Time(m)	Management	Aouther	Journal
Ribociclib	1	72	11	change Palbociclib, again developed; topical tacrolimus and mometasone cream	Chuan Yaw Lee al (22).	Cureus. 2025; 17(5): e83513.
Ribociclib	1	72	2	reduced dose ;hydroxyzine (25 mg nightly), a short course of prednisone (20 mg daily), and loratadine (10 mg daily). 0.1% tacrolimus ointment and phototherapy	John Fernando Montenegro al (17).	Diseases. 2025;13(5):158.
Abemaciclib	1	39	10	cutaneous manifestations were mild	Hanadi Alsatti al (24).	Cureus. 2024;16(4):e57677.
Palbociclib	1	58	10	not experience any discomfort, refused treatment	Shan Gao al (25).	Pathol Oncol Res. 2023:29:1611115.
Ribociclib	(1)	46	3	change Palbociclib, again developed, stopped the therapy; antihistamine (ebastine); topical steroid (clobetasol)	Mariangela Pasqualoni al (21).	Front Oncol. 2023:13:1067264.
	(2)	80	12	discontinue ribociclib		
Ribociclib	1	56	4	Topical immunosuppressive therapy and oral corticosteroids	Alper Türkel al (26).	J Oncol Pharm Pract. 2023: 10781552231156521.
Ribociclib	1	70	8	topical immunosuppressants in combination with oral corticosteroids	Nicolás Silvestre Torner al (16).	Dermatol Pract Concept. 2022; 12(2):e2022045.
Ribociclib	1	78	7	oral mini- pulse betamethasone (5 mg on two consecutive days per week), levocetrizine (5 mg at night) and moisturisers.	Gopikrishnan Anjaneyan al (27).	BMJ Case Rep. 2022;15(4):e248782.
Ribociclib	1	71	5	topical steroids	Baha’ Sharaf al (28).	Clin Cosmet Investig Dermatol. 2022:15:5-10.
Ribociclib	(1)	71	7	ribociclib cessation	On B Chan al (23).	Asia Pac J Clin Oncol. 2022;18(2):e154-e156.
	(2)	54	3			

Based on the present case report and previous relevant cases, we recommend prompt dermatoscopic/Wood’s lamp examination for unexplained pruritus or hypopigmentation during medication. Mild-to-moderate cases may be managed with hormonal/calcineurin inhibitors (as demonstrated in this case) without immediate drug discontinuation. Concurrently, before treatment initiation, patients should receive explicit information regarding this potential adverse reaction with emphasis on early reporting importance. Therapeutic regimens may include topical corticosteroids, topical tacrolimus, UVB phototherapy, or supportive measures such as photoprotection (29). Patients should also be informed of the condition’s benign nature and the necessity for multidisciplinary collaboration (e.g., dermatology, psychiatry, endocrinology, oncology) should any concerns arise.

## 4 Conclusions

Patients with HR-positive metastatic breast cancer who use ribociclib may develop vitiligo skin lesions, and due to individual differences, there may be an incubation period of more than one year as reported in this case. Combined with previous case reports, this reveals that there is a certain connection between ribociclib and the occurrence of vitiligo. Such skin adverse reactions typically do not interfere with the continuation of treatment and are associated with better tumor control and longer survival., and they can also serve as potential biomarkers for evaluating the efficacy of immunotherapy. In the future, further clinical observations and related mechanism studies are needed to confirm this inherent causal relationship, and risk explanations should be provided in advance to patients who are going to use this drug. To help patients understand and cooperate with treatment, thereby improving therapeutic effectiveness and patients’ quality of life.

## Data Availability

The original contributions presented in the study are included in the article/supplementary material. Further inquiries can be directed to the corresponding author.
