# Correction: Vitiligo associated with ribociclib therapy: a rare case report

**DOI:** 10.3389/fonc.2026.1790586

**Published:** 2026-01-26

**Authors:** Zirui Wang, Yuanrui Bai, Rihan Wu, Yihui Liu, Chunhui Dong, Ling Chen

**Affiliations:** 1 Department of Oncology, The First Affiliated Hospital of Xi’an Jiaotong University, Xi’an, China; 2 Health Science Center, Xi’an Jiaotong University, Xi’an, China; 3 Radiotherapy Oncology, People’s Hospital of Ningxia Hui Autonomous Region, Yinchuan, China; 4 Cardiovascular Hospital, Ninth Hospital of Xi’an, Xi’an, China

**Keywords:** ribociclib, vitiligo, CDK4/6 inhibitor, breast cancer, immune-related adverse event

Affiliation “Department of Oncology, The First Affiliated Hospital of Xi’an Jiaotong University, Xi’an, China” was omitted from the paper. This affiliation has now been added for author ZiRui Wang.

The original version of this article has been updated.

